# Correction

**Published:** 1902-02

**Authors:** 


CORRECTION.—By a typographical error in January, 1902, number of the JOURNAL, page 64, "For Sale," third line, reads, "practice averaging twenty thousand dollars per year," when it should read, "practice averaging two thousand dollars per year."

